# Lifestyle vs. pharmacological interventions for healthy aging

**DOI:** 10.18632/aging.102741

**Published:** 2020-01-10

**Authors:** Regula Furrer, Christoph Handschin

**Affiliations:** 1 Biozentrum, University of Basel, Basel, Switzerland

**Keywords:** aging, exercise, metformin, rapamycin, caloric restriction

**Figure 1:**
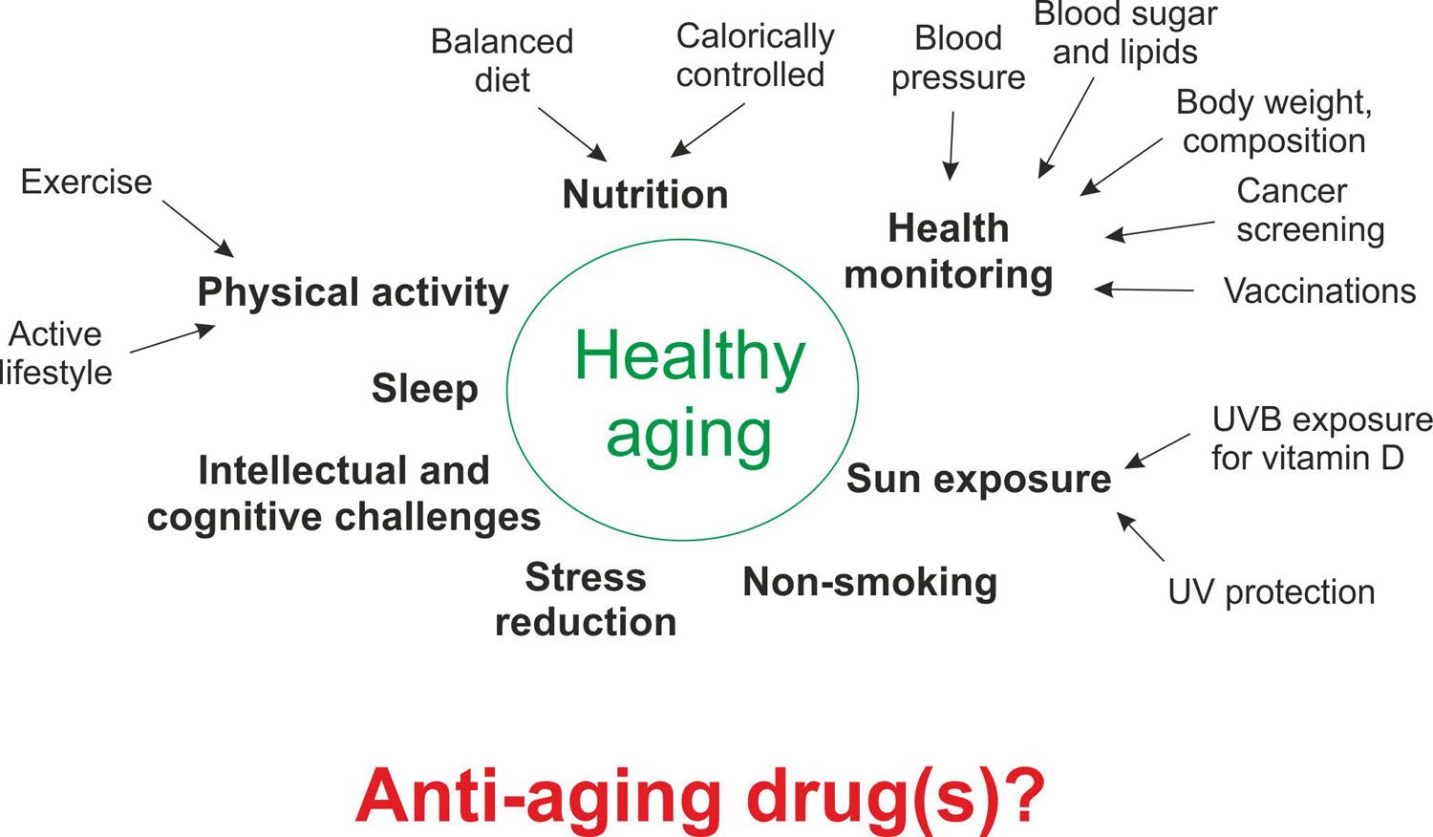
**How to age in a healthy manner.** Examples of behavioral and lifestyle aspects that reduce the risk for developing chronic diseases, help in mitigating pathological events, and decrease morbidity and mortality, thus collectively contributing to healthy aging. At the moment, it is unclear how a single or even multiple pharmacological agents can elicit a similar broad and complex response.

The fountain of youth, the elixir of life, the Philosopher’s stone, or an analogous mythical object to remedy the scourges of aging, has been sought after throughout the history of humankind, up to the present day. In modern times, inventing a drug that prevents the aging-linked decline in organ function, expands the years of life spent in good health, or even increases lifespan promises fame and fortune for the discoverer. Vitamins, anti-oxidants, resveratrol and other alleged sirtuin activators, caloric restriction, nicotinamide adenine dinucleotide (NAD^+^) and its biosynthetic precursors, young blood and growth and differentiation factor 11 (GDF 11), senolytics, rapamycin and rapalogs, metformin as well as numerous other compounds and treatments all were (or still are) considered as the magic bullet for “anti-aging” effects in the last couple of years [1]. However, for most, if not all of them, preclinical results in animal models were difficult to translate to humans, unexpected adverse effects in animals or humans were reported, and/or clinical trials showing any efficacy in healthy young and old individuals are still elusive [1]. Importantly, aging *per se* is not recognized as a disease, and so-called “anti-aging” effects are often difficult to disentangle from disease prevention. For example, it is not entirely clear whether the beneficial outcome of caloric restriction in non-human primates is due to a reduction of numerous diseases observed in control-fed primates (whatever control levels mean in a laboratory context for these animals), or if true “anti-aging” effects were achieved [2]. In stark contrast to the currently proposed putative “anti-aging” drugs, a combination of various lifestyle-based approaches clearly achieves the best epidemiological risk profile for healthy aging, with minimal or no adverse effects (Figure 1). Moreover, some of these approaches, for example exercise training, are not only highly efficient in preventing certain chronic diseases, but also in the treatment of numerous pathologies [3]. However, the molecular basis of the health beneficial effect of exercise remains largely enigmatic. For example, novel protective pathways elicited by exercise training, e.g. via the effect of the central regulator peroxisome proliferator-activated receptor γ coactivator 1α (PGC-1α) on mitochondrial calcium handling, endoplasmic reticulum stress, tubular aggregates and cell death in old muscle, highlight the complexity of the training response and some of the consequences for muscle aging in sedentary or active individuals [4]. In fact, exercise remains the only currently available intervention to mitigate, and even reverse the age-related decline in muscle mass and function, known as sarcopenia. As in other diseases, the usage of anabolic steroids and growth hormones for the treatment of sarcopenia has largely failed, due to lack of efficacy in non-replacement therapies and unacceptable adverse effects. Moreover, since the molecular underpinnings of the etiology and progression of sarcopenia are unknown, a more targeted pharmacological intervention remains elusive. In recent years, the controversial concept of designing and deploying so-called exercise “mimetics”, pharmacological compounds that induce effects similar to those observed after *bona fide* training, has gained traction to circumvent the inherent problem of insufficient mechanistic knowledge [5]. The ongoing broad discussion about exercise “mimetics” to a large extent mirrors that about potential “anti-aging” drugs. In both fields, it is difficult to reconcile how pharmacological modulation of specific pathways would address the complex, pleiotropic, multi-faceted and systemic plasticity observed in exercise adaptation and the aging process, respectively [6]. Intriguingly, besides the overlap in some compounds proposed to work as exercise, caloric restriction “mimetics” and “anti-aging” drugs, such as resveratrol [5], other compounds exert seemingly diametrically opposite effects. For example, the anti-anabolism elicited by putative “anti-aging” drugs such as rapamycin, or the inhibition of insulin and insulin-like growth factor signaling in experimental models, are opposed to the anabolic action that is desired in exercise in general, but most particularly in resistance training. Indeed, the activity of the mammalian target of rapamycin complex 1 (mTORC1) is elevated in resistance-trained muscle, and rapamycin efficiently blocks muscle hypertrophy in certain paradigms. Similarly, metformin and resveratrol impaired beneficial adaptations to endurance exercise in some trials, even though mechanistically, these two compounds could have been expected to initiate endurance training-like effects [1,5]. These observations emphasize the complexity of cellular and organismal adaptation to exercise and aging that impedes the development of pharmacological monotherapies. Thus, the next years will not only reveal whether promising preclinical results with such compounds will stand the test of time in human trials, but also indicate the compatibility with other interventions and treatments. However, as long as data about clinical efficacy and safety of exercise “mimetics” and “anti-aging” drugs are missing (and probably even beyond that), lifestyle-based interventions remain the mainstay approach to minimize the risk for diseases, reduce morbidity and mortality and most importantly, improve healthspan in aging [7]. The old adage “use it or lose it” should thus serve as a reminder that regular physical activity is directly and strongly linked to health in the young and the elderly [8].
